# Risk exposure trade-offs in the ontogeny of sexual segregation in Antarctic fur seal pups

**DOI:** 10.1093/beheco/araa018

**Published:** 2020-03-17

**Authors:** Kayleigh A Jones, Hannah Wood, Jonathan P Ashburner, Jaume Forcada, Norman Ratcliffe, Stephen C Votier, Iain J Staniland

**Affiliations:** 1 Ecosystems Team, British Antarctic Survey, High Cross, Madingley road, Cambridge, UK; 2 College of Life and Environmental Sciences, University of Exeter, Penryn Campus, Penryn, UK

**Keywords:** behavior, early life stages, habitat use, sexual size dimorphism, socialization

## Abstract

Sexual segregation has important ecological implications, but its initial development in early life stages is poorly understood. We investigated the roles of size dimorphism, social behavior, and predation risk on the ontogeny of sexual segregation in Antarctic fur seal, *Arctocephalus gazella*, pups at South Georgia. Beaches and water provide opportunities for pup social interaction and learning (through play and swimming) but increased risk of injury and death (from other seals, predatory birds, and harsh weather), whereas tussock grass provides shelter from these risks but less developmental opportunities. One hundred pups were sexed and weighed, 50 on the beach and 50 in tussock grass, in January, February, and March annually from 1989 to 2018. Additionally, 19 male and 16 female pups were GPS-tracked during lactation from December 2012. Analysis of pup counts and habitat use of GPS-tracked pups suggested that females had a slightly higher association with tussock grass habitats and males with beach habitats. GPS-tracked pups traveled progressively further at sea as they developed, and males traveled further than females toward the end of lactation. These sex differences may reflect contrasting drivers of pup behavior: males being more risk prone to gain social skills and lean muscle mass and females being more risk averse to improve chances of survival, ultimately driven by their different reproductive roles. We conclude that sex differences in habitat use can develop in a highly polygynous species prior to the onset of major sexual size dimorphism, which hints that these sex differences will increasingly diverge in later life.

## Teaser

Sexual segregation is common in the animal kingdom, but how it develops is rarely studied. Small sex differences in habitat use occur in Antarctic fur seal pups as males spend more time in riskier habitats than females. Only the “best” males get to reproduce, so they must take risks to gain strength and social skills in preparation for future fights over access to mates. In contrast, females need to prioritize survival and, hence, prefer safer habitats.

## INTRODUCTION

Sexual segregation can occur across space, time, diet, and behavior and give rise to resource partitioning, which could reduce intraspecific competition (Schoener 1986). However, such segregation may also expose the sexes to different mortality risks (e.g., from human activities), which could lead to biased sex ratios and cause local extinctions (Ruckstuhl and Clutton-Brock 2005). Understanding how sexual segregation develops and how it relates to sex-specific survival can improve our ability to effectively manage habitats and conserve species (Rubin and Bleich 2005; Ruckstuhl and Clutton-Brock 2005; Wearmouth and Sims 2008).

Sexual segregation has predominantly been studied in the adult life stages of a wide range of taxa, including pinnipeds (Staniland 2005; Wearmouth and Sims 2008). Drivers of sexual segregation in adults are thought to relate to several nonmutually exclusive hypotheses, including size dimorphism, social roles (such as the constraints of parental care), and sensitivity to predation risk (Conradt 2005). However, the initial development of sexual segregation is poorly studied. Investigating the hypotheses for sexual segregation in early life stages could reveal valuable insights as individuals have no reproductive commitments (Salton et al. 2019) and sexual size dimorphism is less pronounced.

Sexual size dimorphism is common in polygynous species, whereby males are usually larger than females (Weckerly 1998). The sexual size dimorphism hypothesis states that the sexes have different energetic requirements as the larger sex has a lower mass-specific metabolic rate and higher digestive efficiency than the smaller sex (Ruckstuhl 2007). This proximate cause of sexual segregation could ultimately be driven by males investing more resources into growth as larger males generally compete for mates more successfully (Isaac 2005), whereas females invest more resources into reproduction (Trivers 1972; Clutton-Brock et al. 1982; Reeve and Fairbairn 2001; Schulte-Hostedde et al. 2001). Although sexual size dimorphism is usually minimal in early life stages, the sexes may differ in body composition and metabolic rate, which could affect their resource use (Arnould et al. 1996; Arnould et al. 2001).

The social roles hypothesis proposes that sexes invest in behaviors to prepare for roles required in their reproductive years (Whiteside et al. 2017). Males are generally more active and physically aggressive to compete for mates, whereas females are more passive and risk averse as their social roles relate to protection and parental care (Pellegrini et al. 2005). This may ultimately be driven by the more variable reproductive success in males than females (Darwin 1871). Early life sex differences in behavior occur in African elephants, *Loxodonta* africana, as females remained closer to their mothers, whereas males engaged in more play with unfamiliar peers (Lee 1986). Male mouflon lambs, *Ovis gmelini*, also demonstrated more sexual and agonistic behaviors than females prior to the onset of sexual size dimorphism (Guilhem et al. 2006). These sex differences may develop in additional species in early life stages.

Animals make decisions reflecting trade-offs between predation risk and energetic and social benefits gained by conducting certain activities (Lima and Dill 1990) or selecting particular patches of habitat (Schoener 1971; Mangel and Clark 1986; Willems and Hill 2009). The predation risk hypothesis states that the more vulnerable sex uses safer habitats under the threat of predation (Croft et al. 2004) as a proximate cause of sexual segregation. Females may favor habitats that maximize the safety of offspring, whereas males select higher-risk habitats to maximize energy reserves and growth rates, which could ultimately improve lifetime reproductive success (Main et al. 1996). For example, female house crickets, *Acheta domesticus*, delayed foraging in the presence of shrew odor, whereas males did not respond to the predation risk (Tanis et al. 2018). During reproduction, female little bustards, *Tetrax tetrax*, selected microhabitats in vegetation that balanced shelter with visibility for predator surveillance, whereas males chose suitable structures to be conspicuous for sexual display (Morales et al. 2008). It is poorly known whether these sex differences in risk avoidance emerge in early life stages.

Pinnipeds are an excellent model for studying the ontogeny (development) of sexual segregation. Most land-breeding species demonstrate striking sexual size dimorphism and polygyny in adulthood (Weckerly 1998; Staniland 2005; Wolf et al. 2005), which are suitable characteristics to explore the size dimorphism and social roles hypotheses. Size and social differences may emerge in male and female pups as pups undergo physical and behavioral changes while transitioning from suckling on land to foraging independently at sea (e.g., Luque et al. 2007). Testing the predation risk hypothesis is also appropriate in early life stages as pups are less able to defend themselves against predators and conspecific aggression (Doidge et al. 1984a). Although juvenile males (hereby, independently weaned individuals) travel further at sea than females in several pinniped species (Warren et al. 2006; Leung et al. 2012; Carter et al. 2017), drivers of this segregation remain poorly understood. However, they may relate to constraints imposed by sex differences in body size (Salton et al. 2019).

Antarctic fur seals are one of the most in-depth studied otariids and adults sexually segregate in foraging distribution (Staniland 2005; Staniland and Robinson 2008). They are highly polygynous, so reproductive success varies substantially among males, which hold harems of 1–27 females at a time (McCann 1980) and will fight to the death to gain access to mates. Only the most competitive males will reproduce; for example, out of 600 pups, a quarter were fathered by only 12 males (Hoffman et al. 2003). The size dimorphism seen in adults occurs from birth as males are (on average) born 0.5 kg heavier than females (Payne 1979) and grow faster than females during the lactation period (Kerley 1985). Socialization is essential in male otariid pups as they frequently play fight (rarely observed in females) and mimic copulatory behavior to prepare for their reproductive roles in later life (Bartholomew 1959; Gentry 1974; Arnold and Trillmich 1985; Warren et al. 2006).

Antarctic fur seal pups must balance trade-offs between developmental needs and exposure to risk. At Bird Island, South Georgia, there is a clear delineation in habitats: beaches, water, and tussock grass. Beaches and water provide opportunities for socialization and learning as the open spaces allow pups to interact and form social groups and water facilitates play in young seals (e.g., Wilson 1974; Wilson and Jones 2018). However, pups are at risk of injury and death from predatory seabirds, fighting territorial males, rebuffs from other seals, and harsh weather conditions (Bartholomew 1959; Doidge et al. 1984a). Areas of tussock grass, *Poa flabellata*, are elevated, densely vegetated regions that provide shelter from these risks but fewer opportunities for social interaction. Indeed, mothers preferentially suckle in safer less disturbed areas of the tussock grass as soon as the pup is physically capable of completing the journey from the pupping beach (Doidge et al. 1984a).

During the 4-month lactation period, mothers alternate foraging at sea (2–11 days) with suckling their pups ashore (1–2 days) (Forcada and Staniland 2009), so pups are alone for the majority of this time. This represents one of the shortest lactation periods among otariids, during which pups must not only grow but also acquire a range of skills to maximize their chances of surviving and breeding in future. There have been few studies on Antarctic fur seal pups other than those related to their growth (Doidge et al. 1984b; Lunn et al. 1993) and acquisition of diving skills (McCafferty et al. 1998), so the development of their behavior and any differences between the sexes are currently unknown.

We studied the habitat use of preweaned Antarctic fur seal pups to test hypotheses for the ontogeny of sexual segregation in early life stages. Using movement data from pups tracked using GPS loggers and counts of pups found on the beach and in the tussock grass, we hypothesized that: 1) female pups have a higher association with tussock grass areas than males as they are more risk averse; 2) male pups travel further at sea than females toward the end of lactation as sexual size dimorphism becomes more pronounced; and 3) the ultimate drivers of this sexual segregation relate back to male and female reproductive roles.

## METHODS

### Ethical statement

The procedures in this study were reviewed and approved by the British Antarctic Survey Animal Ethics and Welfare Review Body (AWERB). Procedures adhered to Association for the Study of Animal Behaviour (ASAB) guidelines, Animal Research: Reporting of In Vivo Experiments (ARRIVE) guidelines, and legal requirements of the South Georgia Government. The behavioral response of pups was predictable (based on on-going pup monitoring at the colony) and no pups were injured during handling procedures. It should be noted that the mortality rate of GPS-tracked pups was less than the population average during the study period.

### Population-level sex differences

Antarctic fur seal pups were captured annually at Main Bay, Bird Island, South Georgia (54.010° S, 38.059° W), as part of a long-term monitoring program. One hundred pups were selected (by convenience sampling), 50 on the beach and 50 in the tussock grass, each month in January, February, and March annually from 1989 to 2018. Each pup was captured by hand, measured, sexed (by examination of genitalia), and weighed to the nearest 100 g (using a hand-held spring balance).

### Individual-level sex differences

**Figure 1:**
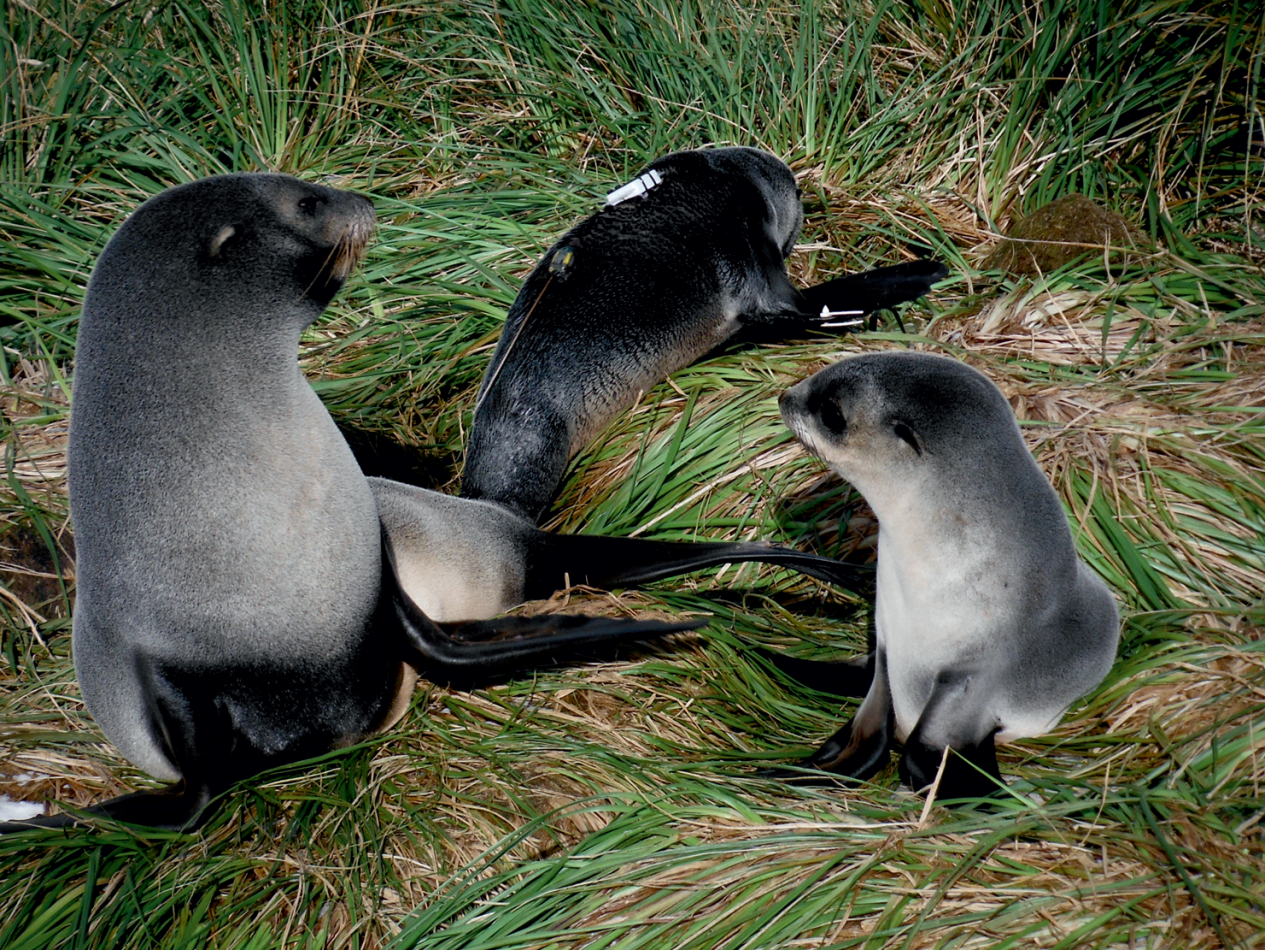
Antarctic fur seal pup deployed with a GPS logger on the upper back, radio transmitter on the lower back, and ID tag on the right fore flipper at Bird Island, South Georgia (Photo: Hannah Wood).

Thirty-five Antarctic fur seal pups, 19 males and 16 females, were GPS-tracked from the beach habitat at Freshwater beach, Bird Island, South Georgia (54.009° S, 38.052° W) between December 2012 and April 2013. To identify individuals, Dalton jumbo roto ID tags were attached to each pup’s fore flippers. Pups were sexed, measured, weighed, and equipped with a GPS logger (i-gotU GT-600; 37 g; 46 × 41.5 × 14 mm) and a radio transmitter (Sirtrack V2G-152A; 16 g, 40 × 20 × 10 mm; Figure 1). The radio transmitter was glued with quick-set epoxy resin onto the fur on each pup’s lower back on the central dorsal line. A rectangle of mesh fabric (40 × 20 mm) was glued between the scapula, and GPS loggers were fixed with cable ties to this mesh, allowing the easy interchange of units when their battery charge had depleted (after ~13 days). GPS loggers were programmed to record locations every 5 min and pups were recaptured and weighed every 3.74 ± 0.076 days until the pups weaned or died. GPS loggers and radio transmitters attached to weaned pups would have detached from their fur during the next molt.

#### GPS data processing

Speed and distance thresholds for each pup were obtained using the 99th percentiles found by the distSpeed function in the diveMove package (Luque 2017) in the software R (R Core Team 2017). These thresholds were used in a speed filter (based on Austin et al. 2003) that removes erroneous locations in a three-stage process as described by Staniland et al. (2012). We then used Correlated Random Walk Library (CRAWL) (Johnson 2017) to fit a state-space model to the data to account for uncertainty in GPS fixes (Johnson et al. 2008) and estimate locations evenly spaced in time (every 5 min). Gaps in data (caused by loss of battery life prior to tag change) were taken into account by removing specified sections of time. Since GPS signals could not be received effectively in water, best-fit tracks sometimes indicated that pups moved over substantial headlands, when they had evidently swum around land. In these cases, tracks were adjusted to prevent implausible movements and CRAWL was rerun to represent the best-fit tracks more accurately. Pups that suffered premature mortality (mostly caused by starvation at the beginning of the lactation period) were not included in analyses as the duration of tracking was short.

### Data analysis

#### Pup growth

On a population level, to test whether pup growth significantly differed between the sexes with month (indicating stage of pup development), location (beach and tussock grass habitats), and year (to determine any long-term trends from 1989 to 2018) in monitored pups at Bird Island, we used average pup mass as the response variable in a general linear model (GLM). We also tested whether growth rates differed between male and female pups (from January to March) in years when environmental conditions were good and poor in a GLM using gentoo penguin, *Pygoscelis papua*, breeding success (ratio of chicks to nests) at Bird Island (1989–2018) as an indicator of krill availability. Gentoo penguin breeding success was chosen as an appropriate indicator as it is highly sensitive and positively correlated with the proportion of krill in the diet (Waluda et al. 2017), and krill dominates the diet of Antarctic fur seals in the South Atlantic (Forcada and Staniland 2009).

On an individual level, to determine the general trend in mass of male and female GPS-tracked pups with age during the 2012–2013 breeding season, we used pup mass as the response variable in a generalized additive mixed model (GAMM; suitable for nonlinear relationships) using the mgcv package in R (Wood 2017). We specifically used a Gaussian error family and identity link function, with age nested within pup ID as a random factor to account for individual variability. To obtain more accurate mass estimates related to each pup’s growth (and not the meal mass of milk consumed), we fitted a generalized additive model to the mass data for each individual pup to smooth regular fluctuations in mass according to whether pups had suckled. We, then, extracted the modeled mass each day for each individual pup, which we used as an explanatory variable (for pup growth) in further analyses.

#### Pup habitat use

To test for sexual segregation in pups between beach and tussock grass habitats at the population level, as well as determine any changes in sexual segregation between months and years, we analyzed the pup monitoring data using sex ratio as the response variable in a generalized linear model with a binomial error and logit link function.

To investigate sexual segregation in habitat use at the individual level, we tested whether sex differences occurred between GPS-tracked pups with age and mass using a simplified habitat classification (tussock grass or other) based on multispectral light wavelengths from an aerial image of Bird Island overlaid with the best-fit tracks. For each pup, we determined the proportion of time that pups spent in the tussock grass each day, which we used as the response variable in a GAMM using the mgcv package in R (Wood 2017). We used a Beta error family (suitable for continuous proportional data bounded by 0 and 1; Thomas et al. 2017) and we specified pup ID as a random effect to account for individual variability. Because pup habitat use during the early lactation period is heavily influenced by the mother, the analysis was divided into two sections based on pup ages, that is, 20–40 days (when mothers suckled their pup on the pupping beach) and 41–120 days (when all mothers had led their pup to a new suckling location in the tussock grass). We used 120 days of age as the cutoff point to reduce bias in the analysis because six males and only two females were tracked after this age.

#### Pup trips at sea

GPS-tracked pup movements were classed as “trips” if pups ventured at sea further than 300 m away from the mean coordinate of all pup GPS locations (located near the pupping beach). Start and end times of trips were determined according to when pups had left and returned to the pupping beach using the “TimeManager” plug-in (Graser and Alexiou 2011) in QGIS (QGIS Development Team 2017). We calculated the duration and maximum distance traveled from the pupping beach for each trip. Trip metrics were only analyzed for trips taken up to 120 days of age.

GAMMs, implemented using mgcv, were used to test whether the trip distance and trip duration significantly differed between sexes with age and mass. The trip number was nested within pup ID as a random effect to account for deviance among repeated trips made by the same individuals. The maximum trip distance traveled was log transformed to improve model fit. To determine whether the proportion of time that trips occurred at night differed between sexes with age and mass, we assigned each observation to day time or night time (according to sunrise and sunset times) and, then, used a GAMM with a Beta error family and specified pup trip number nested within trip ID as a random effect.

For each analysis, we used Akaike information criterion (AIC) to assess model uncertainty by comparing competing models (Symonds and Moussalli 2011). We included all possible interaction terms in candidate models, including tensor product interactions in GAMMs (Wood 2017). We selected the best-fit model for each analysis according to the lowest AIC. If best-fit models differed by only two AIC, we selected the simplest model with all explanatory variables significantly associated with the response variable. Best-fit models were also checked using the dredge function in the MuMIn package in R, which ranks all candidate models by their fit (Barton 2017). All means are reported with one standard error unless otherwise stated.

### Data overview

#### Pup monitoring

The sample size for the number of data points for sex ratios of pups during the monitoring period was 180, accounting for the sex ratio in beach and tussock grass habitats over 3 months each year for 30 years (1989–2018). The sample size for the number of data points for average pup mass during the monitoring period was 360, accounting for average pup mass of males and females in each habitat over 3 months for 30 years (1989–2018).

#### Pup tracking

Thirty-five pups (16 females and 19 males) were GPS-tracked but six pups died during the study period (Supplementary Table S1a). This mortality rate of 17.1% was lower than the overall pup mortality rate at Bird Island (23.3%) during the 2012–2013 pupping season. A sample size of 29 pups (13 females and 16 males that survived; Supplementary Table S1b) was, therefore, used in the analyses. This included 24 pups (10 females and 14 males) tracked between 20 and 40 days of age and all 29 pups tracked between 41 and 120 days of age.

## RESULTS

### Sex differences in growth

#### Pup monitoring

**Figure 2:**
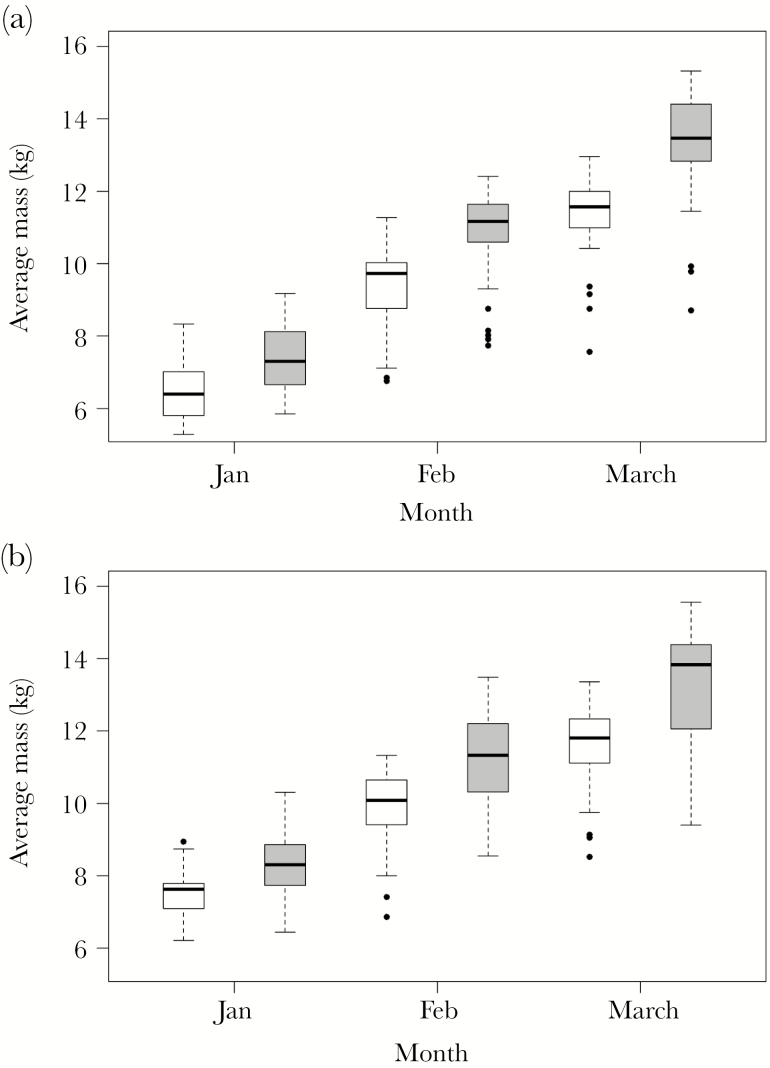
Boxplots showing the mass of female (white) and male (gray) Antarctic fur seal pups on the beach (a) and in the tussock grass (b) from long-term monitoring at Bird Island, South Georgia: 100 pups were selected, sexed, and weighed, 50 on the beach and 50 in tussock grass, each month in January, February, and March each year from 1989 to 2018 (sample size of 360 data points for average pup mass in total). Bold lines are the median values, boxes give the interquartile range (IQR) and whiskers give 1.5 × IQR.

Mass of monitored pups was significantly associated with the interaction between sex, habitat and month, and with year (GLM: adjusted *R*^2^ = 0.79, *F*_8, 351_ = 170.3, *P* < 0.0001; sex:habitat:month *F*_2, 351_ = 4.3, *P* = 0.01; year *F*_1, 351_ = 52.9, *P* < 0.0001; Supplementary Table S2). Specifically, male pups were heavier than females, pups weighed in the tussock grass (where their mass was affected by meal mass of milk consumed) were heavier than those weighed on the beach, and pups gained mass as they developed from January to March (Figure 2). Sexual size dimorphism became more pronounced as pups developed: on average, males were 0.87, 1.37, and 1.78 kg heavier than females in January, February, and March, respectively. Pup mass of both sexes generally declined by 1.44 ± 0.15 kg from 1989 to 2018. Sex was an important factor in the model as the difference in AIC between the best-fit model and candidate model excluding sex was 113.7.

Pup mass each month was significantly associated with sex and gentoo penguin breeding success (used as an indicator of food availability; GLM: adjusted *R*^2^ = 0.80, *F*_6, 353_ = 240.6, *P* < 0.0001; sex:month:gentoo breeding success *F*_2, 353_ = 3.7, *P* = 0.03; Supplementary Table S3). In years when environmental conditions were inferred as good (gentoo penguin breeding success = 1.6 chicks on average per nest), males grew faster than females and were 2.23 ± 0.22 kg heavier than females by March (Supplementary Figure S4). In years when environmental conditions were inferred as poor (gentoo penguin breeding success = 0 chicks on average per nest), males were only 1.25 ± 0.22 kg heavier than females by March (Supplementary Figure S4). The difference in AIC between the best-fit model and candidate model excluding gentoo penguin breeding success was 84.3.

#### Pup tracking

**Figure 3:**
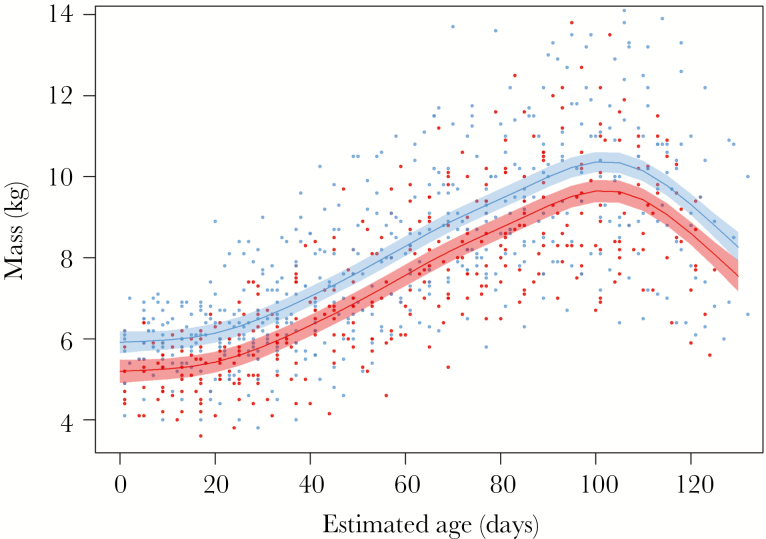
GAMM showing the general trend in mass of 16 male (blue) and 13 female (red) GPS-tracked Antarctic fur seal pups with estimated age between December 2012 and April 2013. Points indicate pup mass at each individual weighing, lines indicate modeled averages, and shaded areas indicate standard error.

In GPS-tracked pups, mass ranged from 3.6 to 13.8 kg in females and 3.8 to 16.5 kg in males. Mass gain was significantly associated with sex and age (GAMM: *R*^2^ = 0.56, s[age by sex] *F*_7.1, 873.9_ = 343.3, *P* < 0.0001; Supplementary Table S5). Male pups remained 0.71 kg heavier than female pups on average, but the trend in mass was the same for both sexes: pups gained mass at an average of 0.04 kg/day between 0 and 100 days of age and lost mass thereafter at 0.05 kg/day (Figure 3). The difference in AIC between the best-fit and second best-fit model (which excluded sex) was 2.3.

### Sex differences in habitat use

#### Pup monitoring

**Figure 4:**
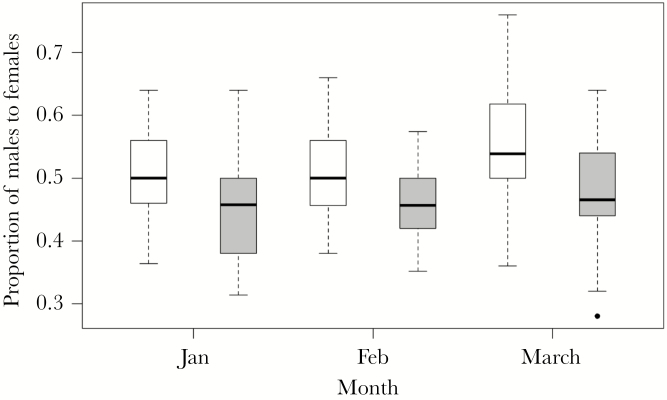
Boxplot showing the proportion of male to female Antarctic fur seal pups during long-term monitoring at Bird Island, South Georgia: 100 pups were selected, sexed, and weighed, 50 on the beach and 50 in tussock grass, each month in January, February, and March each year from 1989 to 2018 (sample size of 180 data points for sex ratios in total). Bold lines are the median values, boxes give the interquartile range (IQR), and whiskers give 1.5 × IQR.

Sex differences in habitat use were apparent in monitored pups at Bird Island during 1989–2018. Proportion of male to female pups was significantly associated with habitat, month, and year (generalized linear model: pseudo *R*^2^ = 0.18, *F*_3, 176_ = 0.82, *P* = 0.49; habitat *P* < 0.0001, month *P* = 0.04, year *P* < 0.001; Supplementary Table S6). Addressing each factor, males were more likely to occur on the beach than females (mean proportion of males to females ± SE = 0.52 ± 0.01) and females were more likely to occur in the tussock grass than males (mean proportion of males to females ± SE = 0.46 ± 0.01). Proportion of males to females marginally increased in both habitats from 0.48 ± 0.01 in January to 0.50 ± 0.01 in March (Figure 4). Proportion of males to females also significantly increased over the study period from a mean ratio of 0.46 ± 0.01 in 1989 to 0.52 ± 0.01 in 2018. The second best-fit model (within two AIC of the selected model) included the same explanatory variables as the best-fit model but also included an interaction between month and year (which had no significant effect).

#### Pup tracking

**Figure 5:**
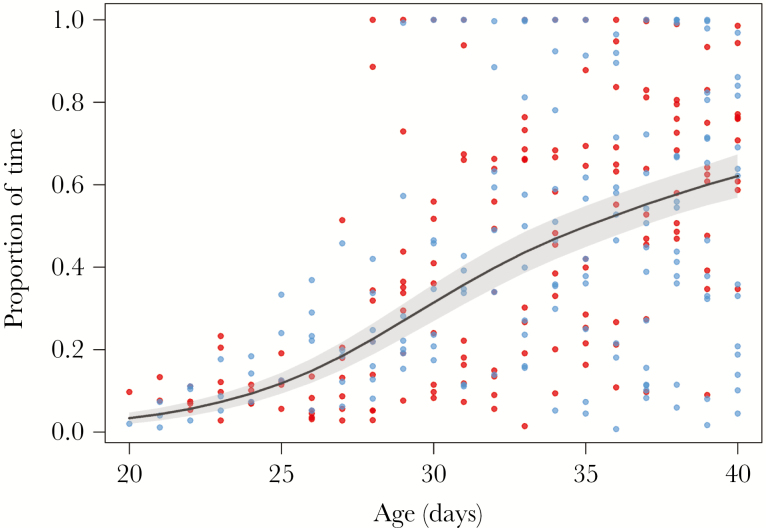
GAMM showing the proportion of time that 10 female (red) and 14 male (blue) GPS-tracked Antarctic fur seal pups spent in the tussock grass between an estimated 20 and 40 days of age. Points indicate proportion of time spent in the tussock grass each day by individuals, line indicates modeled average, and shaded area indicates standard error.

From 20 to 40 days of age, 24 GPS-tracked pups (5 out of 29 pups were not tracked over this time) spent a progressively higher proportion of time in the tussock grass and the best-fit model indicated no significant difference between the sexes (GAMM: *R*^2^ = 0.26, s[age] *F*_2.5, 313.5_ = 64.3, *P* < 0.0001; Supplementary Table S7). Pups spent an average of 3.4 ± 1.4% of time in the tussock grass at 20 days of age and 62.1 ± 5.3% of time in the tussock grass at 40 days of age (Figure 5). The second best-fit model was within two AIC of the best-fit model and included sex as an additional explanatory variable (which had no significant effect).

**Figure 6:**
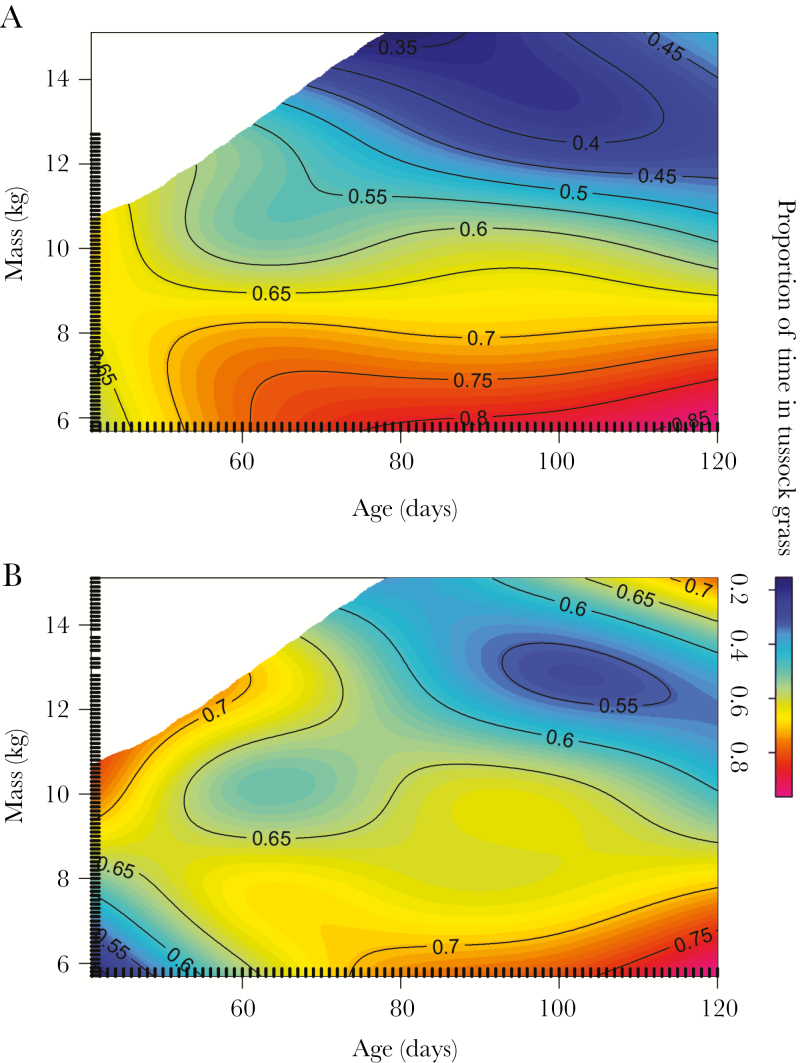
GAMM showing the proportion of time that (a) 13 female and (b) 16 male GPS-tracked Antarctic fur seal pups spent in the tussock grass between an estimated 41 and 120 days of age. Rugs (tick marks inside plot) indicate locations of all data points.

Between 41 and 120 days of age, the proportion of time that GPS-tracked pups spent in the tussock grass was significantly associated with pup mass and sex, as well as the interaction between pup mass and age (GAMM: *R*^2^ = 0.04, s[mass]; *F*_1, 1829.1_ = 25.7, *P* < 0.0001; s[mass, by sex]; *F*_1, 1829.1_ = 25.7, *P* < 0.001; ti[mass, age]; *F*_6.8, 1829.1_ = 4.8, *P* < 0.0001; Supplementary Table S8). Specifically, the proportion of time that females spent in the tussock grass was closely associated with their mass (small females spent most time in the tussock grass), whereas the proportion of time that males spent in the tussock grass was more variable with mass (Figure 6). Both sexes generally spent less time in the tussock grass as they developed, but lightweight pups (less than 8 kg) spent a high proportion of time in the tussock grass toward the end of lactation (Figure 6). Although the effect size of this best-fit model was small, the model had the lowest AIC and explained the most variation out of candidate models. The model excluding sex had a higher AIC (difference of 2.2) and explained less variation (*R*^2^ = 0.02).

Regarding habitat use of pups that died during the study, three pups (two males and one female) remained on the beach for the majority of time during tracking but died of starvation between 17 and 23 days of age. Three additional pups used the beach, tussock, and bay habitats during tracking but died between 39 and 52 days of age: one male and one female died of starvation, whereas the other female drowned in a bog.

### Sex differences in movements

#### Ontogeny of movements

Both male and female GPS-tracked pups undertook progressively longer, more distant trips out at sea (from the pupping beach) as they developed. Pups generally returned to previously explored haul-out sites before extending their trip distances. However, occasionally, pups made sudden long-distance trips, such as to the main island of South Georgia, with no prior experience of the area. The first female and male pups that traveled more than 300 m in distance from the mean GPS point near the pupping beach were 48 and 49 days old, respectively. Between 0 and 120 days of age, 522 trips were recorded in total: 222 by 13 females and 300 by 16 males.

**Figure 7:**
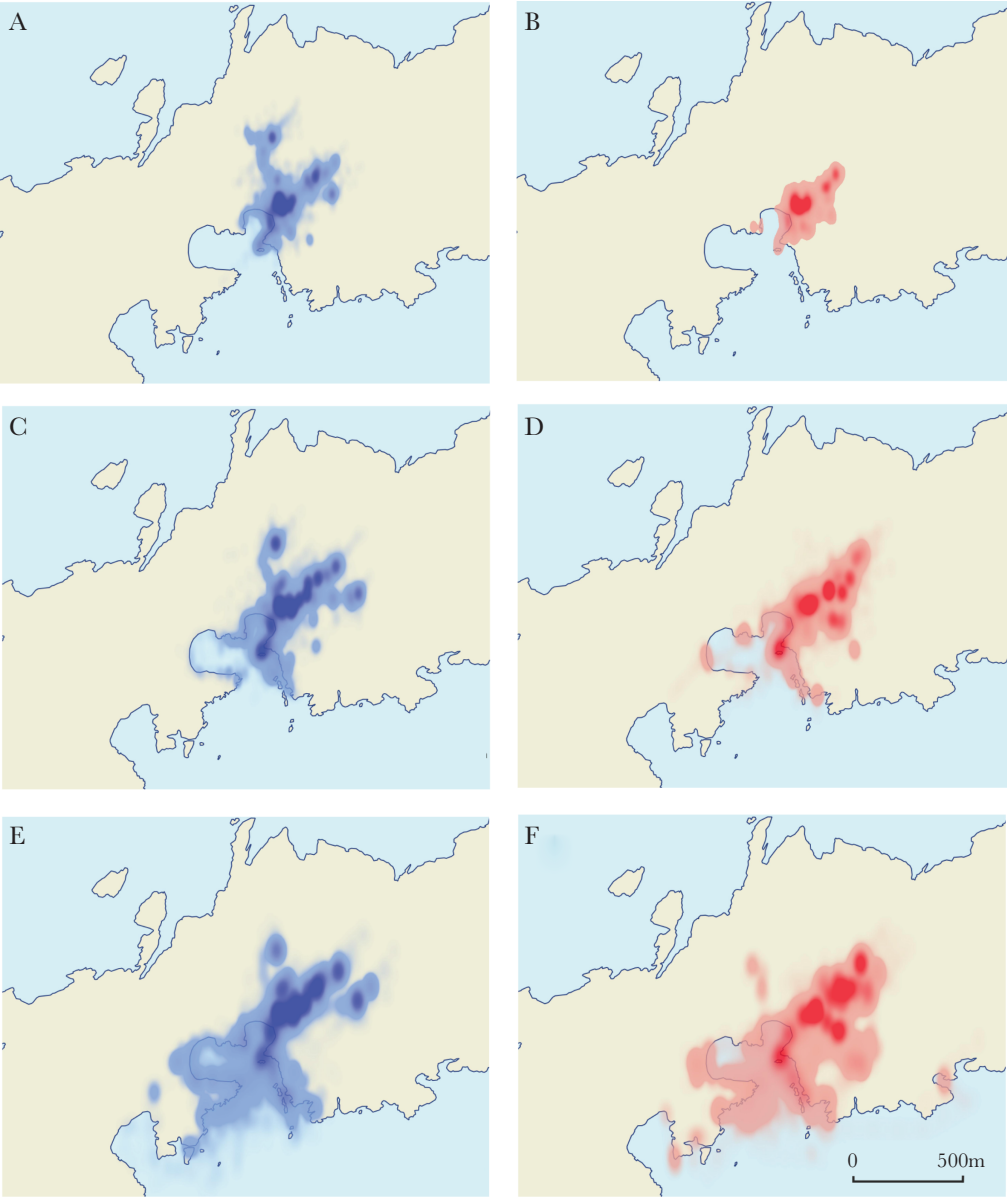
Heat maps with 99% of cumulative points showing ontogeny of pup movements and use of land at Bird Island (beige) and sea (light blue) from male (blue) and female (red) Antarctic fur seal pups: (a) 14 males and (b) 10 females between ages 20 and 40 days; (c) 14 males and (d) 10 females between ages 41 and 60 days; (e) 15 males and (f) 13 females between ages 61 and 80 days.

Between 20 and 40 days of age, pups mainly spent time on the pupping beach in established suckling locations within the tussock grass or on the immediate coastline (Figure 7a,b). Between 41 and 60 days of age, pups had established suckling locations in the tussock grass and traveled to coasts both within and outside Freshwater Bay (Figure 7c,d). They further extended their ranges between 61 and 80 days of age (Figure 7e,f).

**Figure 8:**
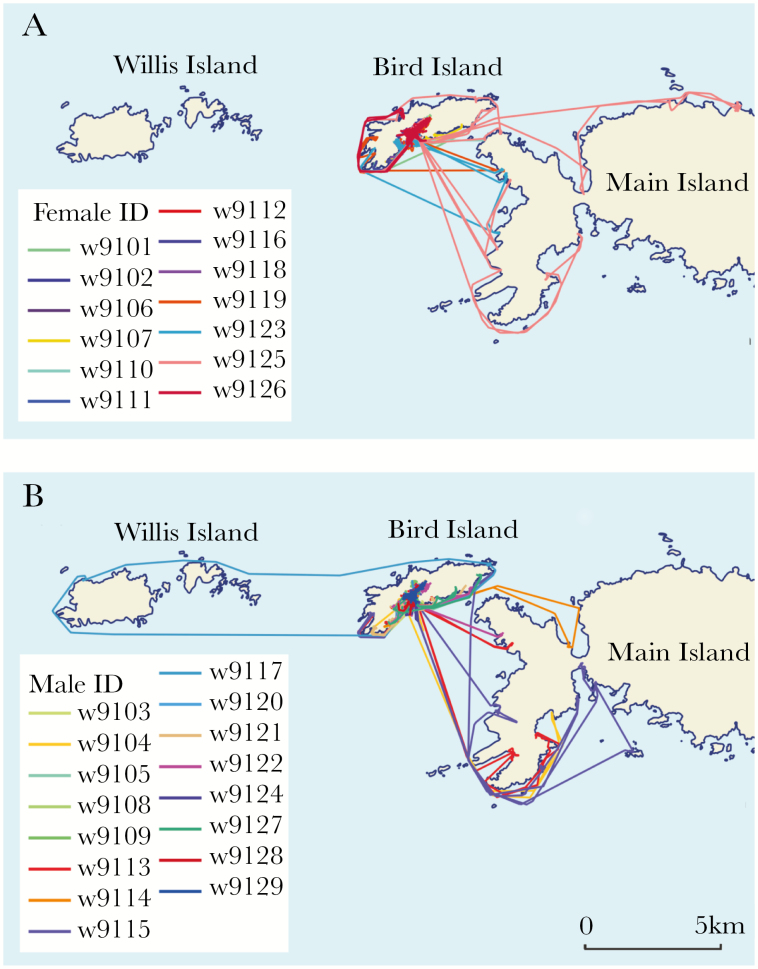
GPS tracks of (a) 13 female and (b) 16 male Antarctic fur seal pups between 80 and 120 days of age. Lines represent minimum distance traveled between haul-out locations and colors indicate different individuals.

Pups explored the coasts of Bird Island and surrounding islands between 81 and 120 days of age (Figure 8). They generally returned to their suckling locations immediately after returning from their trips. One female (w9125) traveled 11 289 m away from the pupping beach at 89 days of age and explored the north-west coast of the main island of South Georgia. This trip distance was 6.5 times greater than the average distance traveled by pups at this age, and the outlier was removed from trip analyses. The female pup also traveled to the south-west of the main island, which was not frequented by any other female pup. Her suckling location was located in the tussock grass behind the research station—notably closer to the breeding beach than those of other female pups. Only one pup (male w9117) traveled to Willis Island (west of Bird Island).

#### Trips at sea

**Figure 9:**
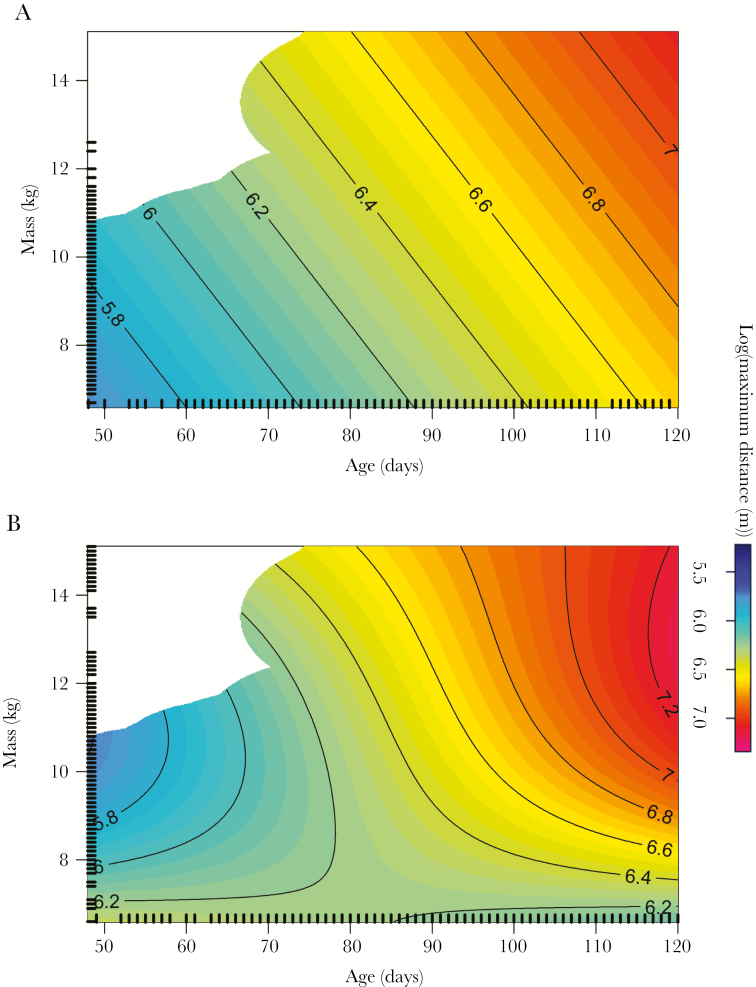
GAMM showing log of maximum distance traveled by female and male GPS-tracked Antarctic fur seal pups according to their age and mass based on (a) 221 trips by 13 female pups and (b) 300 trips by 16 male pups. Rugs (tick marks inside plot) indicate locations of all data points.

Maximum distance traveled by GPS-tracked pups on trips at sea was significantly associated with age, mass, and the interaction between age and mass with sex (GAMM: *R*^2^ = 0.21, s[age] *F*_1, 515.8_ = 80.1, *P* < 0.0001; s[mass] *F*_1, 515.8_ = 8.42, *P* = 0.004; ti[age, mass, by sex]: *F*_2.17, 515.8_ = 4.7, *P* = 0.01; Supplementary Table S9). Specifically, both sexes traveled further at sea as they aged and gained mass, but males traveled further than females toward the end of the lactation period (Figure 9). The second best-fit model was within two AIC of the best fit model and had the same structure with an additional interaction between mass and sex.

Trip duration was significantly associated with the interaction between age and mass, but effect size was small (GAMM: *R*^2^ = 0.03, s[age] *F*_1, 514.9_ = 9.2; ti[age, mass] *P* = 0.003; *F*_4.1, 514.9_ = 6.0, *P* < 0.0001; Supplementary Table S10). Trip duration increased during development, particularly toward the end of the lactation period (Supplementary Figure S11). The second best-fit model was within two AIC and also included an interaction between mass and sex (which had no significant effect) and an interaction between age, mass, and sex.

The proportion of time that trips occurred at night was significantly associated with sex and age, but effect size was also small (GAMM: *R*^2^ = 0.03, s[age] *F*_1, 518_ = 8.5, *P* = 0.004; sex *P* = 0.006; Supplementary Table S12). Between 50 and 120 days of age, the proportion of time that pups spent on trips during the night increased from 25.6 ± 2.3 % to 39.9 ± 3.5 % in males and 31.6 ± 3.1 % to 49.4 ± 4.2 % in females (Supplementary Figure S13). The difference in AIC between the best-fit model and second best-fit model (which included sex and mass) was 71.0.

## DISCUSSION

This is one of the few studies to show that small sex differences in habitat use can develop in a highly polygynous species prior to weaning. We found that sexual segregation began to develop in Antarctic fur seal pups at Bird Island, South Georgia, both on land and at sea: 1) analysis of pup counts in beach and tussock grass habitats (from 1989–2018) suggested that female pups had a slightly higher association with tussock grass habitats than males. Small sex differences were found in tussock grass use by GPS-tracked pups (after 40 days of age), which also depended on pup mass—lightweight females spent the most time in the tussock grass. 2) Pups traveled further out to sea as they developed, but males traveled slightly further than females toward the end of the lactation period. We use these findings to investigate the predation risk, social roles, and size dimorphism hypotheses as they relate to early life sexual segregation.

### Size dimorphism

Sexual size dimorphism was present in pups during the monitoring period and in GPS-tracked pups as males remained heavier than females on average. Monitoring data suggested that sexual size dimorphism became more pronounced from January to March, but this trend did not occur in GPS-tracked pups during the 2012–2013 breeding season. In favorable conditions, male Antarctic fur seal growth rates often exceed that of females (Lea et al. 2006; Vargas et al. 2009; present study). This is thought to reflect the need for male pups to attain a relatively large size, which can improve breeding success in later life (Doidge and Croxall 1989; Isaac 2005). When foraging conditions are poor, pup growth is constrained by the mother’s milk supply. Our results suggest that foraging conditions during the 2012–2013 breeding season were poor, supported by lower pup growth rates (44 vs. 79 g/day; Doidge and Croxall 1989), a decline in mass after 100 days of age, and an elevated mortality rate (23.3% compared with a 5-year mean of 14%). Monitoring data also showed that pups in the tussock grass were heavier than those on the beach as they had likely suckled more recently (and had more milk in their stomachs).

### Sexual segregation in habitat use

Initially, pups are led by their mothers from suckling on the beach to the safer elevated region of tussock grass (Doidge et al. 1984a). Therefore, there was no sex bias in habitat use of GPS-tracked pups in their first 40 days of age as tussock grass use was strongly influenced by the decisions of mothers. Slight sex differences occurred in tussock grass use between 41 and 120 days of age: lightweight females generally spent more time in the tussock grass than heavy females and males of the same mass. This sex difference was supported by long-term monitoring data as males were more commonly found on the beach and females in the tussock grass.

At Bird Island, beach and tussock habitats vary dramatically in risk exposure. Beaches and water provide the best opportunities for pup social interaction. The open spaces allow pups to form social groups, whereas water facilitates playful behavior in young seals (e.g., Wilson 1974; Wilson and Jones 2018). However, the beach is highly populated and pups are at increased risk of injury and death. Adult males fight when attempting to defend, obtain, or expand their territories (McCann 1980), often trampling pups, disturbing the colony and causing mothers and pups to separate (Doidge et al. 1984a). Juvenile animals regularly harass pups, and adult females will bite pups (other than their own) that get too close (Doidge et al. 1984a). Giant petrels, *Macronectes spp.*, brown skuas, *Stercorarius antarcticus,* and sheathbills, *Chionis spp*, also attack pups. Sheathbills peck wounds (Doidge et al. 1984a), which can lead to mortality, whereas giant petrels prey on weak pups or drive swimming pups into deeper water to exhaust and drown them. Beaches are also exposed to wind, rain, snow, and waves, which entail high thermoregulatory and energetic costs. Tussock grass provides shelter and protection from these hazards. Our findings suggest that larger pups are better able to cope with dangers on the beach as they are less vulnerable to predation, hypothermia, and starvation than smaller pups. However, males appear more risk prone than females of the same mass, which indicates that sex differences in social behavior also influence habitat use.

Optimality Theory proposes that animals only perform behaviors if life-history benefits exceed costs (Harcourt 1991a). Generally, males have a higher propensity for risk-taking and dangerous behavior than females (Wrangham 1999). Males tend to be more competitive, energetic, and physically aggressive to develop fighting skills and dominance (Clutton-Brock 1983; Beier and McCullough 1990). Social play in young males can involve mounting and fighting, which mimics adult behavior and enhances skills needed to compete for mates in later life (e.g., Gentry 1974; Smith 1982; Harcourt 1991b). Females are generally less active and aggressive, as their social roles relate better to protecting and provisioning offspring (Pellegrini 2004). They tend to be more risk averse and may avoid vigorous behavior by males (Harpers and Sanders 1975; Pellegrini 2004). Indeed, male Steller sea lion, *Eumetopias jubatus*, and Galapagos fur seal, *Arctocephalus galapagoensis*, pups play fight more frequently than females (Gentry 1974; Arnold and Trillmich 1985). These behavioral differences are driven by perinatal androgens (Goldfoot et al. 1984; Hines and Kaufman 1994; Archer and Lloyd 2002).

Animals must assess reward with the cost of aggregating in areas with high mortality risk (Schoener 1971; Mangel and Clark 1986; Willems and Hill 2009). Play behaviors can be particularly costly. For example, the majority of South American fur seal pups, *Arctocephalus australis*, predated on by Southern sea lions, *Otaria flavescens*, at a colony in Peru were distracted by play at the time of the attack (Harcourt 1991a), suggesting that play came at a cost of vigilance. Despite the risk of early mortality, which is the most severe cost to an animal, pups continued to play in high-risk areas of the beach (Harcourt 1991a). Also, cow elk, *Cervus canadensis,* increased vigilance and decreased feeding in the presence of wolves, whereas bulls (the larger sex) showed neither response—likely unable to pay the associated foraging costs (Winnie and Creel 2007). These sex differences in risk avoidance could explain the small sex differences in Antarctic fur seal pup habitat use.

Male Antarctic fur seal pups may spend slightly more time in the high-risk beach environment to socialize and play fight to gain musculature, experience, and social skills, whereas females spend slightly more time in the safer tussock grass to improve chances of survival. Larger pups are also less vulnerable to injury and predation, so larger males are the most risk prone, whereas small females are the most risk averse. Similar patterns in habitat use have also been reported in guppies, *Poecilia reticulata*, which assorted in size and sex under risk of predation from the Trinidadian pike cichlid, *Crenicichla frenata* (Croft et al. 2004). Males (the brightly colored and more vulnerable sex) preferred safer waters by the riverbank, whereas cryptically colored females preferred deeper (and riskier) waters, and both sexes were longer in mean body length in deeper waters (Croft et al. 2004). Our findings indicate that body size, social roles, and predation risk may all contribute to small sex differences in pup habitat use.

Although our results only explained a low proportion of variation, we were measuring behaviors in a wild population and were unable to control for other influencing factors, such as mother fitness, pup genetics, pup health, time between suckling bouts, location of suckling area (i.e., distance from the pupping beach), weather conditions, and changes in predator assemblages. Despite these limitations, we demonstrated the influence of sex and size on risk exposure at both an individual and a population level.

### Sexual segregation in trip metrics

Trip duration at sea did not significantly differ between male and female pups as it is constrained by their mothers’ foraging decisions. Although pups are free to explore between suckling bouts, they generally return to their suckling locations before their mothers return from foraging. Our findings suggest that mothers invest the same amount of time suckling male and female Antarctic fur seal pups, which provides support that there is no sex bias in milk consumption (Arnould et al. 1996).

Light level is an important factor in decision-making because it affects the visual abilities of predators and prey (Lima and Dill 1990). Female Antarctic fur seal pups spent a slightly greater proportion of time on trips during the night than males. Although the effect size was low, this result may reflect small sex differences in behavior: females spending slightly more time on trips at night to reduce risk by avoiding aggressive and dangerous attacks by predatory seabirds. This sex difference in trip metric has also been recorded in adults during the mating season as females foraged more frequently during the night time than males, potentially, to reduce diving costs by exploiting prey that vertically migrate to the surface at night (Staniland and Robinson 2008).

Pups traveled further at sea as they aged and gained mass, but males traveled slightly further than females of the same mass toward the end of lactation. As pups developed, they gained the appropriate physiology, locomotor skills, and experience to swim further while their mothers foraged (Salton et al. 2019). Pups also acquire a more slender body shape and larger fore flippers (Luque et al. 2007) and their blood volume and blood oxygen stores increase, which improves their diving capabilities (McCafferty et al. 1998) and subsequent swimming skills (Bowen et al. 1999; Jørgensen et al. 2001). These skills enable pups to catch small prey items approaching weaning age, indicated by traces of crustaceans in their scats (Doidge et al. 1986).

Sex differences in trip distances may be driven by social roles, predation risk, and body dimorphism (Salton et al. 2019). In highly social polygynous mammals, males tend to be more dispersive than females (Greenwood 1980), so males may travel further to prospect sites and evaluate the best foraging areas and potential future mating opportunities, whereas females will return to their natal site to breed and provision offspring. Female pups may also be more risk averse and make shorter distance trips to improve chances of survival. Travelling at sea is risky as small naïve pups explore new regions with different predators (e.g., orcas, *Orcinus orca*, and sixgill sharks, *Hexanchus sp.*) and unpredictable environmental conditions. Pups risk drowning, getting lost, and starving. Males may be more risk prone, gaining experience exploring potential foraging sites to maximize growth rates, as polygynous males are in an energetic race to maximize body condition to compete for mates (Main and Toit 2005). A similar trade-off has been documented in adolescent male long-tailed macaques, *Macaca fascicularis,* which become mostly solitary during several months of high fruit abundance; this increases predation risk but maximizes foraging intake, enabling them to grow rapidly and improve mating opportunities (Watts 2005).

Male and female Antarctic fur seal pups also differ in body composition and physiology. Males direct more energy toward lean tissue growth and females toward accumulating fat stores (Arnould et al. 1996). Females, therefore, have a higher mass-specific metabolic rate (Arnould et al. 2001) and are less efficient at gaining mass than males (Guinet et al. 1999). Females may travel shorter distances to conserve energy or they may be less capable of long trips at sea, as swimming entails energetic costs of physical movement and thermoregulation (and smaller pups have higher costs of maintaining body temperature in frigid waters). Because juvenile otariids with larger body sizes can have higher mass-specific oxygen stores (e.g., Fowler et al. 2007), males may be better divers than females. Their hearts and lungs also constitute a greater proportion of total body mass (Payne 1979). Males may, therefore, develop the physiological capabilities, including greater strength and breath-holding abilities, to travel further than females of the same mass toward the end of lactation—enabling them to take more risks at sea. These findings indicate that sexual segregation will become more pronounced after weaning. Indeed, Warren et al. (2006) found that weaned male Antarctic fur seals traveled substantially further from their birth sites (at Bird Island) than females (maximum distances recorded: 900 and 400 km, respectively).

### Environmental implications

Sexual segregation in Antarctic fur seal pups may depend on the nature of the mortality risk (e.g., predator assemblage and seal density), habitat composition, and prey availability. Pups are more prone to injury and death at beaches with high seal densities (Doidge et al. 1984a), and habitat composition and availability of refuge areas can shape antipredator behaviors (Wcisel et al. 2015). Sexual segregation may be more pronounced in years with high prey availability as sexual size dimorphism will be more extreme. The fact that we detected small sex differences in habitat use even in a year with poor prey availability and minimal sexual size dimorphism suggests that sexual segregation could be a vital aspect of the Antarctic fur seals’ life-history strategy. Sex differences in habitat use may manifest differently in pups of other otariid species (e.g., Galápagos sea lions, *Zalophus wollebaeki*; Piedrahita et al. 2014) as a result of different lactation strategies and predictability of environmental conditions.

### Drivers of behavior

Kernaléguen et al. (2016) proposed that size dimorphism and breeding constraints do not directly drive sexual segregation in otariids. However, our findings suggest that the initial development of sexual segregation in Antarctic fur seals may be explained by underlying drivers of behavior, resulting from intense sexual selection pressures. These sexual selection pressures and the coercive behavior of males on females may have originally evolved after sexual size dimorphism and polygyny (Krüger et al. 2014; Cassini et al. 2020). Because reproductive success is more varied in males than females (Darwin 1871), male Antarctic fur seals must gain social skills (e.g., by play fighting) and build muscle mass early in life if they are to successfully reproduce in future. Sexual size and body dimorphism, therefore, occurs even in pups, and male pups may be more risk prone than females, resulting in small sex differences in habitat use.

## CONCLUSIONS

Investigating the drivers of sexual segregation is key to understanding how the sexes may respond differently to mortality risk. Sexual segregation has predominantly been studied in adults, but studying ontogeny of sexual segregation in early life stages can reveal how this phenomenon initially develops. Our study has improved understanding of these processes by showing that body dimorphism, social roles, and predation risk may all contribute to small sex differences in habitat use and exploratory behavior of Antarctic fur seal pups by influencing risk exposure trade-off decisions. Males may be more risk prone and invest in behaviors to prepare for intense competition for mates, whereas females (particularly small females) may be more risk averse to improve chances of survival, which is ultimately driven by their different reproductive roles. Our findings hint that sex differences in behavior will increasingly diverge in later life, resulting in more pronounced sexual segregation. Life-history strategies play fundamental roles in the ontogeny of sexual segregation and studying sexual segregation in additional species in the initial life stages could underpin species-specific drivers of this phenomenon. Such insights are crucial to understand the requirements of each sex for survival to inform habitat management and species conservation efforts.

## FUNDING

This work was supported by the Natural Environment Research Council Capability Fund and the Natural Environment Research Council Great Western Four+ Doctoral Training Partnership (NE/L002434/1).

We thank the British Antarctic Survey zoological field assistants and biologists who contributed to the long-term data set by monitoring Antarctic fur seals at Bird Island, South Georgia, from 1989 to 2018. We also thank Dr Devin Johnson for his support using Correlated Random Walk Library (CRAWL), as well as two anonymous reviewers for their useful feedback.

Data accessibility: Analyses reported in this article can be reproduced using the data provided by Jones et al. (2020).

## Supplementary Material

araa018_suppl_Supplementary_Material

## Notes

H. Wood is now at Zoological Society London, Regent’s Park, London, NW1 4RY, UK; J. Ashburner is now at Xodus Group, 50 Huntly St, Aberdeen AB10 1RS, UK
