# Supplementary material for: Risk exposure trade-offs in the ontogeny of sexual segregation in Antarctic fur seal pups
Source: Behav Ecol. 2020 Mar 17;31(3):719–30. doi: 10.1093/beheco/araa018 (PMC7303822; doi:10.1093/beheco/araa018)
Supplement: araa018_suppl_Supplementary_Material [file araa018_suppl_supplementary_material.docx]

**Supplementary Material**

**Risk Exposure Trade-offs in the Ontogeny of Sexual Segregation in**

**Antarctic Fur Seal Pups**

**Jones KA, Wood H, Ashburner J, Forcada J, Ratcliffe N, Votier SC, Staniland IJ.**

**Supplementary Table S1a.** Details of GPS-tracked Antarctic fur seal pups that died (a) and survived (b) during the study period in the 2012 – 2013 pupping season at Bird Island, South Georgia. *Full days of missing data caused by tag failure: w9104=12/03/2013; w9105=27/03/2018 – 29/03/2018; w9108=24/03/2013 – 26/03/2013; w9109=13/02/2013 – 23/02/2013; w9110=09/02/2013 – 23/02/2013; w9111= 22/03/2013 – 24/03/2013; w9118=19/01/2013 – 02/02/2013; w9127=12/03/2013.

| **Pup ID** | **Sex** | **First tracking obervation** | **Last tracking observation** | **Tracking duration (days)** | **Min–max mass (kg)** | **Date pup died** | **Observation** |
| --- | --- | --- | --- | --- | --- | --- | --- |
| **atp10** | M | 27/12/2012 | 29/12/2012 | 3 | 3.9**–**4.9 | 29/12/2012 | starved, blood in rectum |
| **atp11** | F | 05/01/2013 | 16/01/2013 | 12 | 4.2**–**6.3 | 16/01/2013 | starved |
| **atp13** | M | 21/12/2012 | 25/12/2012 | 4 | 6.5**–**8 | 28/12/2012 | starved |
| **atp18** | F | 27/12/2012 | 31/12/2012 | 5 | 6**–**7.4 | 31/12/2012 | starved, blood in rectum |
| **atp29** | M | 03/01/2013 | 22/01/2013 | 19 | 5.2**–**7.4 | 22/01/2020 | starved |
| **atp38** | F | 10/01/2013 | 29/01/2013 | 20 | 4.8**–**8.3 | 03/02/2013 | drowned in bog |

**Supplementary Table S1b.**

| **Pup ID** | **Sex** | **First tracking observation** | **Last tracking observation** | **Tracking duration (days)** | **Min and max mass (kg)** | **Total**  **number of trips** | **Max trip duration (mins)** | **Max trip distance (m)** |
| --- | --- | --- | --- | --- | --- | --- | --- | --- |
| **w9101** | F | 24/12/2012 | 29/03/2013 | 96 | 4.2**–**11.6 | 17 | 3,600 | 1,947 |
| **w9102** | F | 24/12/2012 | 04/03/2013 | 71 | 4.7**–**9.5 | 15 | 1,650 | 1,949 |
| **w9103** | M | 15/02/2013 | 06/04/2013 | 51 | 3.8**–**10.5 | 18 | 1,020 | 1,682 |
| **w9104** | M | 24/12/2012 | 25/03/2013 | 92-1*=91 | 5.2**–**11.3 | 19 | 2,465 | 8,215 |
| **w9105** | M | 24/12/2012 | 07/04/2013 | 105-3*=101 | 4.9**–**11.4 | 16 | 2,555 | 2,702 |
| **w9106** | F | 24/12/2012 | 22/03/2013 | 89 | 4.5**–**11.9 | 14 | 1,930 | 839 |
| **w9107** | F | 27/12/2012 | 01/04/2013 | 96 | 4.2**–**10.5 | 19 | 1,635 | 1,717 |
| **w9108** | M | 10/01/2013 | 07/03/2013 | 57-3*=54 | 5.8**–**12.2 | 13 | 730 | 1,429 |
| **w9109** | M | 27/12/2012 | 11/03/2013 | 75-11*=64 | 5.2**–**11.1 | 6 | 2,010 | 502 |
| **w9110** | F | 27/12/2012 | 22/03/2013 | 86-15*=71 | 4.5**–**12.2 | 17 | 990 | 2,321 |
| **w9111** | F | 03/01/2013 | 06/04/2013 | 94-2*=92 | 5**–**10.8 | 6 | 865 | 532 |
| **w9112** | F | 03/01/2013 | 14/03/2013 | 71 | 5.4**–**9.5 | 26 | 775 | 840 |
| **w9113** | M | 03/01/2013 | 19/04/2013 | 107 | 5.8**–**11.3 | 29 | 2,415 | 7,965 |
| **w9114** | M | 15/01/2013 | 31/03/2013 | 76 | 5.4**–**16.5 | 25 | 1,955 | 5,768 |
| **w9115** | M | 14/01/2013 | 19/04/2013 | 96 | 5.8**–**13.5 | 26 | 5,540 | 9,428 |
| **w9116** | F | 20/02/2013 | 02/04/2013 | 42 | 3.8**–**8.1 | 18 | 1,690 | 2,174 |
| **w9117** | M | 03/01/2013 | 07/04/2013 | 95 | 5.2**–**12.9 | 19 | 3,835 | 12,821 |
| **w9118** | F | 05/01/2013 | 28/03/2013 | 83-15*=68 | 5**–**10.5 | 15 | 1,950 | 1,462 |
| **w9119** | F | 15/02/2013 | 26/03/2013 | 40 | 3.6**–**9.6 | 23 | 2,055 | 3,324 |
| **w9120** | M | 10/01/2013 | 08/03/2013 | 58 | 5.2**–**11 | 13 | 1,340 | 901 |
| **w9121** | M | 05/01/2013 | 28/03/2013 | 83 | 5.9**–**14.1 | 20 | 5,960 | 2,169 |
| **w9122** | M | 05/01/2013 | 29/03/2013 | 84 | 6.2**–**13.2 | 21 | 2,595 | 3,333 |
| **w9123** | F | 05/01/2013 | 19/03/2013 | 74 | 5.6**–**13.8 | 14 | 4,735 | 4,651 |
| **w9124** | M | 14/01/2013 | 06/04/2013 | 83 | 4.5**–**8.4 | 32 | 620 | 1,029 |
| **w9125** | F | 10/01/2013 | 08/04/2013 | 89 | 4.8**–**12.7 | 28 | 3,260 | 11,289 |
| **w9126** | F | 21/02/2013 | 10/04/2013 | 49 | 4.5**–**9.9 | 10 | 4,640 | 2,588 |
| **w9127** | M | 10/01/2013 | 16/04/2013 | 97-1*=96 | 4.7**–**13.5 | 17 | 2,700 | 2,315 |
| **w9128** | M | 10/01/2013 | 07/04/2013 | 88 | 5.9**–**10.2 | 18 | 830 | 907 |
| **w9129** | M | 27/02/2013 | 08/04/2013 | 41 | 5-7.6 | 8 | 390 | 472 |

**Supplementary Table S2.** Model comparisons to study the effects of sex, month and habitat (beach or tussock grass) and their interactions on average Antarctic fur seal pup mass at Bird Island, South Georgia, during pup monitoring from 1989 **–** 2018. AIC: Akaike’s information criterion; ΔAIC: difference in AIC between candidate model and best-fit model; R^2^: proportion of variance explained by predictors; n: number of observations of the response variable. Model parameters are shown for the best-fit model.

| **Predictors** | **AIC** | **ΔAIC** | **R^2^** | **n** |
| --- | --- | --- | --- | --- |
| sex + habitat + month + year + sex:habitat + sex:month + sex:habitat:month | 1112.6 | 0 | 0.79 | 360 |
| sex + habitat + month + year + sex:habitat + sex:month + sex:year + sex:habitat:month | 1113.4 | 0.8 | 0.79 | 360 |
| sex + habitat + month + year + sex:habitat + sex:month + sex:year + sex:habitat:month + sex:habitat:year | 1116.9 | 4.3 | 0.79 | 360 |
| sex + habitat + month + year + sex:habitat + sex:month | 1117.3 | 4.7 | 0.79 | 360 |
| habitat + month + year + habitat:month | 1226.3 | 113.7 | 0.71 | 360 |
|  |  |  |  |  |
|  | **Value** | **SE** | **t-value** | **p-value** |
| Intercept | 103.42 | 13.64 | 7.58 | < 0.0001 |
| sex (male) | 0.42 | 0.44 | 0.96 | 0.34 |
| habitat (tussock) | 1.38 | 0.44 | 3.13 | 0.001 |
| month | 2.40 | 0.14 | 16.65 | <0.0001 |
| year | -0.05 | 0.01 | -7.28 | <0.0001 |
| sex (male): habitat (tussock) | -0.01 | 0.62 | -0.02 | 0.98 |
| sex (male): month | 0.49 | 0.20 | 2.39 | 0.02 |
| sex (female): habitat (tussock): month | -0.40 | 0.20 | -1.96 | 0.05 |
| sex (male): habitat (tussock): month | -0.45 | 0.20 | -2.19 | 0.03 |

**Supplementary Table S3.** Model comparisons to study the effects of sex, month and gentoo penguin breeding success (as an indicator of prey availability) on average Antarctic fur seal pup mass at Bird Island, South Georgia, during pup monitoring from 1989 – 2018. AIC: Akaike’s information criterion; ΔAIC: difference in AIC between candidate model and best-fit model; R^2^: proportion of variance explained by predictors; n: number of observations. Model parameters are shown for the best-fit model.

| **Predictors** | **AIC** | **ΔAIC** | **R^2^** | **n** |
| --- | --- | --- | --- | --- |
| sex + month + gentoo breeding success + sex:month + sex:month:gentoo breeding success | 1093.6 | 0 | 0.80 | 360 |
| sex + month + sex:month | 1177.9 | 84.3 | 0.75 | 360 |
| month + gentoo breeding success +  month:gentoo breeding success | 1215.9 | 122.3 | 0.72 | 360 |
|  |  |  |  |  |
|  | **Value** | **SE** | **t-value** | **p-value** |
| Intercept | 4.39 | 0.37 | 12.0 | <0.0001 |
| sex (male) | 0.42 | 0.30 | 1.36 | 0.17 |
| month | 2.02 | 0.18 | 11.4 | <0.0001 |
| gentoo breeding success | 0.58 | 0.32 | 1.79 | 0.07 |
| sex(m):month | 0.28 | 0.18 | 1.58 | 0.12 |
| sex(f):month:gentoo breeding success | 0.20 | 0.16 | 1.23 | 0.22 |
| sex(m):month:gentoo breeding success | 0.40 | 0.16 | 2.52 | 0.01 |

**Supplementary Figure S4.** General linear model showing the growth of female (red) and male (blue) Antarctic fur seal pups during long-term monitoring at Bird Island, South Georgia: 100 pups were selected, sexed and weighed, 50 on the beach and 50 in tussock grass, each month in January, February and March each year from 1989 – 2018. Points show average mass of 100 pups, dotted lines show average growth when krill availability is predicted poor (indicated by gentoo penguin breeding success of 0 chicks per nest) and dashed lines show pup growth when krill availability is predicted good (indicated by gentoo penguin breeding success of 1.6 chicks per nest). Shaded areas indicate standard error.
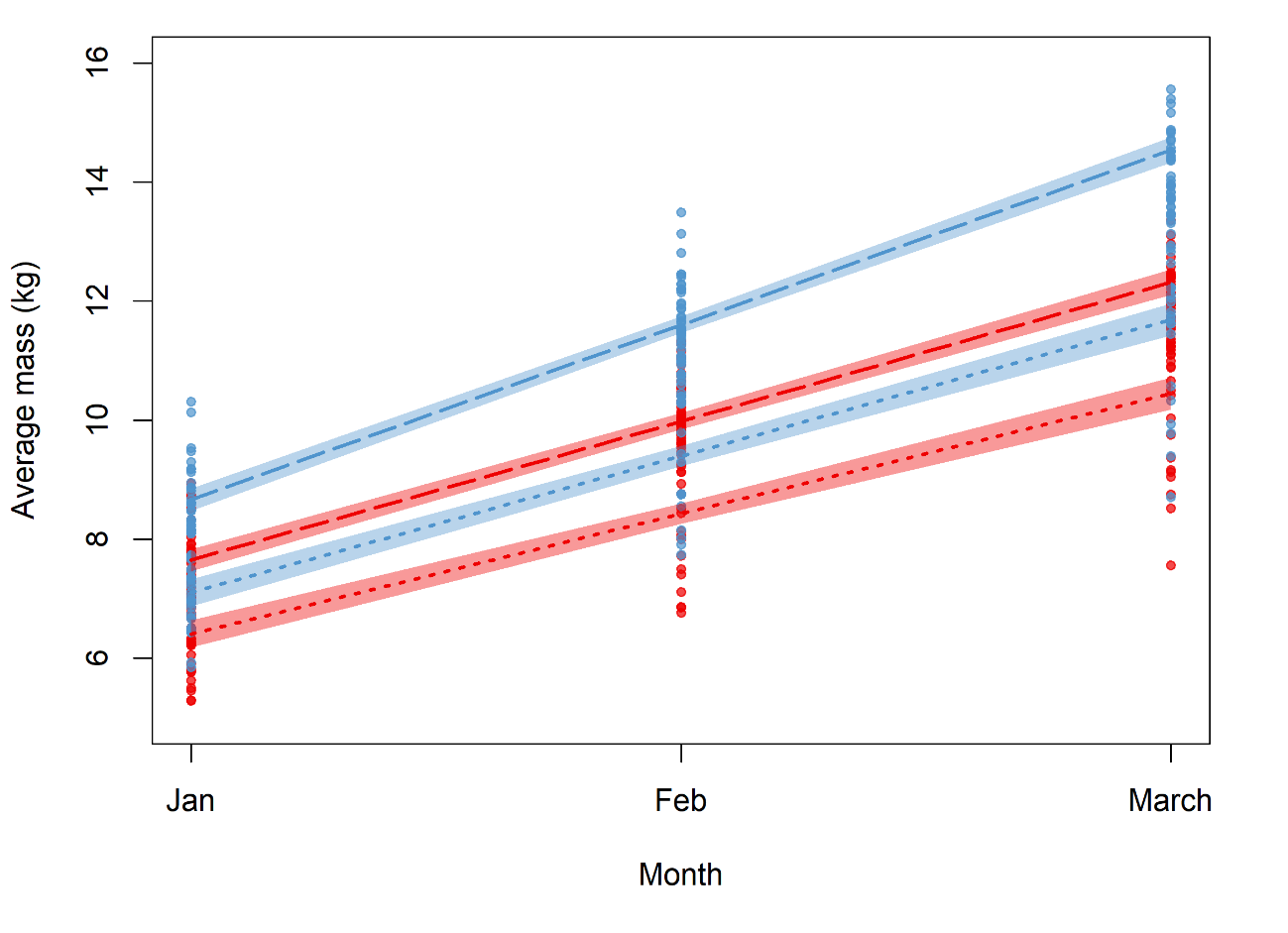


**Supplementary Table S5.** Model comparisons to study the effects of sex, age and their interactions on mass of GPS-tracked Antarctic fur seal pups at Bird Island, South Georgia. AIC: Akaike’s information criterion; ΔAIC: difference in AIC between candidate model and best-fit model; R^2^: proportion of variance explained by the predictors; n (N) number of observations of the response variable and number of individuals respectively. Model parameters are shown for the best-fit model.

| **Predictors** | **AIC** | **ΔAIC** | **R^2^** | **n (N)** |
| --- | --- | --- | --- | --- |
| sex + s(age) | 2562.8 | 0 | 0.56 | 883 (29) |
| s(age) | 2565.1 | 2.3 | 0.53 | 883 (29) |
| sex + s(age by sex) | 2587.1 | 24.3 | 0.56 | 883 (29) |
|  |  |  |  |  |
| *Parametric coefficients* | **Value** | **SE** | **t-value** | **p-value** |
| Intercept | 7.39 | 0.25 | 29.1 | <0.0001 |
| sex (male) | 0.72 | 0.34 | 2.09 | 0.04 |
| *Approximate significance of smooth terms* | **edf** | **Ref.df** | **F** | **p-value** |
| s(age) | 7.06 | 7.06 | 343.3 | <0.0001 |
|  |  |  |  |  |

**Supplementary Table S6.** Model comparisons to study the effects of habitat (beach or tussock grass), month (January to March), year and their interactions on the proportion of male to female Antarctic fur seal pups at Bird Island, South Georgia, during pup monitoring from 1989 **–** 2018. AIC: Akaike’s information criterion; ΔAIC: difference in AIC between candidate model and best-fit model; pseudo R^2^: proportion of variance explained by predictors; n: number of observations of the response variable. Candidate models with ΔAIC < 5 are presented. Model parameters are shown for the best-fit model.

| **Predictors** | **AIC** | **ΔAIC** | **pseudo R^2^** | **n** |
| --- | --- | --- | --- | --- |
| habitat + month + year | 1000.1 | 0 | 0.18 | 180 |
| habitat + month + year + month:year | 1001.3 | 1.2 | 0.19 | 180 |
| habitat + month + year + habitat:month | 1001.7 | 1.6 | 0.18 | 180 |
| habitat + month + year + habitat:year | 1001.9 | 1.8 | 0.18 | 180 |
| habitat + month + year + habitat:month + month:year | 1002.9 | 2.8 | 0.19 | 180 |
| habitat + month + year + month:year + location:year | 1003.1 | 3.0 | 0.19 | 180 |
| habitat + year | 1003.2 | 3.1 |  | 180 |
| habitat + month + year + habitat:month + habitat:year | 1003.4 | 3.3 | 0.19 | 180 |
| habitat + month + year + habitat:month + habitat:year + month:year | 1004.6 | 4.5 | 0.19 | 180 |
|  |  |  |  |  |
|  | **Value** | **SE** | **t-value** | **p-value** |
| Intercept | -18.6 | 5.3 | -3.54 | <0.001 |
| habitat (tussock) | -0.22 | 0.05 | -4.79 | <0.0001 |
| month | 0.06 | 0.03 | 2.08 | 0.04 |
| year | 0.01 | 0.003 | 3.54 | <0.001 |

**Supplementary Table S7.** Model comparisons to study the effects of sex, smooth function of mass, smooth function of age, and their interactions on tussock grass use by GPS-tracked Antarctic fur seal pups from 20 – 40 days of age at Bird Island, South Georgia. AIC: Akaike’s information criterion; ΔAIC: difference in AIC between candidate model and best-fit model; R^2^: proportion of variance explained by the predictors; n (N) number of observations of the response variable and number of individuals respectively. Candidate models with ΔAIC < 5 are presented. Model parameters are shown for the best-fit model.

| **Predictors** | **AIC** | **ΔAIC** | **R^2^** | **n (N)** |
| --- | --- | --- | --- | --- |
| s(age) | 1005.0 | 0 | 0.260 | 317 (24) |
| sex + s(age) | 1006.2 | 1.2 | 0.258 | 317 (24) |
| sex + s(mass) | 1006.2 | 1.2 | -0.126 | 317 (24) |
| s(age) + s(age by sex) | 1008.7 | 3.7 | 0.267 | 317 (24) |
| s(age) + ti(age, mass) | 1009.2 | 4.2 | 0.250 | 317 (24) |
|  |  |  |  |  |
| *Parametric coefficients* | **Value** | **SE** | **t-value** | **p-value** |
| Intercept | -0.53 | 0.19 | -2.79 | 0.006 |
| *Approximate significance of smooth terms* | **edf** | **Ref.df** | **F** | **p-value** |
| s(age) | 2.46 | 2.46 | 64.27 | <0.0001 |

**Supplementary Table S8.** Model comparisons to study the effects of sex, smooth function of mass, smooth function of age, and their interactions on tussock grass use by GPS-tracked Antarctic fur seal pups from 41 – 120 days of age at Bird Island, South Georgia. AIC: Akaike’s information criterion; ΔAIC: difference in AIC between candidate model and best-fit model; R^2^: proportion of variance explained by the predictors; n (N) number of observations of the response variable and number of individuals respectively. Candidate models with ΔAIC < 5 are presented. Model parameters are shown for the best-fit model.

| **Predictors** | **AIC** | **ΔAIC** | **R^2^** | **n (N)** |
| --- | --- | --- | --- | --- |
| s(mass) + s(mass by sex) + ti(age, mass) | 5650.1 | 0 | 0.041 | 1839 (29) |
| s(mass) + ti(age, mass) | 5652.3 | 2.2 | 0.022 | 1839 (29) |
| s(age) + s(mass) + s(mass by sex) + ti(age, mass) | 5654.4 | 4.3 | 0.038 | 1839 (29) |
| s(mass) + s(mass by sex) | 5654.8 | 4.7 | 0.030 | 1839 (29) |
|  |  |  |  |  |
| *Parametric coefficients* | **Value** | **SE** | **t-value** | **p-value** |
| Intercept | 0.67 | 0.08 | 7.91 | <0.0001 |
| *Approximate significance of smooth terms* | **edf** | **Ref.df** | **F** | **p-value** |
| s(mass) | 1 | 1 | 25.69 | <0.0001 |
| s(mass by sex) | 1 | 1 | 11.11 | <0.001 |
| ti(age, mass) | 6.90 | 6.90 | 4.84 | <0.0001 |

**Supplementary Table S9.** Model comparisons to study the effects of sex, smooth function of mass, smooth function of age, and their interactions on trip distance of GPS-tracked Antarctic fur seal pups at Bird Island, South Georgia. AIC: Akaike’s information criterion; ΔAIC: difference in AIC between candidate model and best-fit model; R^2^: proportion of variance explained by the predictors; n (N) number of observations of the response variable and number of individuals respectively. Candidate models with ΔAIC < 5 are presented. Model parameters are shown for the best-fit model.

| **Predictors** | **AIC** | **ΔAIC** | **R^2^** | **n (N)** |
| --- | --- | --- | --- | --- |
| s(age) + s(mass) + ti(age, mass by sex) | 769.8 | 0 | 0.209 | 521 (29) |
| s(age) + s(mass by sex) + ti(age, mass by sex) | 769.1 | 0.9 | 0.207 | 521 (29) |
| s(age) + ti(age, mass) | 773.8 | 4 | 0.187 | 521 (29) |
|  |  |  |  |  |
| *Parametric coefficients* | **Value** | **SE** | **t-value** | **p-value** |
| Intercept | 6.36 | 0.03 | 204.2 | <0.0001 |
| *Approximate significance of smooth terms* | **edf** | **Ref.df** | **F** | **p-value** |
| s(age) | 1 | 1 | 80.09 | <0.0001 |
| s(mass) | 1 | 1 | 8.42 | 0.004 |
| ti(age, mass by sex) | 2.17 | 2.17 | 4.67 | 0.009 |

**Supplementary Table S10.** Model comparisons to study the effects of sex, smooth function of mass, smooth function of age, and their interactions on trip duration of GPS-tracked Antarctic fur seal pups at Bird Island, South Georgia. AIC: Akaike’s information criterion; ΔAIC: difference in AIC between candidate model and best-fit model; R^2^: proportion of variance explained by the predictors; n (N) number of observations of the response variable and number of individuals respectively. Candidate models with ΔAIC < 5 are presented. Model parameters are shown for the best-fit model.

| **Predictors** | **AIC** | **ΔAIC** | **R^2^** | **n (N)** |
| --- | --- | --- | --- | --- |
| s(age) + ti(age, mass) | 1181.5 | 0 | 0.030 | 521 (29) |
| s(age) + s(mass by sex) + ti(age, mass by sex) | 1183.4 | 1.9 | 0.043 | 521 (29) |
| s(age) + s(mass) + ti(age, mass by sex) | 1183.7 | 2.2 | 0.042 | 521 (29) |
| s(age) + s(age by sex) + ti(age, mass) | 1184.7 | 3.2 | 0.028 | 521 (29) |
| s(age) + s(mass) + ti(age, mass) | 1185.4 | 3.9 | 0.030 | 521 (29) |
| s(mass)+ ti(age, mass) | 1186.4 | 4.9 | 0.033 | 521 (29) |
|  |  |  |  |  |
| *Parametric coefficients* | **Value** | **SE** | **t-value** | **p-value** |
| Intercept | 6.13 | 0.10 | 62.4 | <0.0001 |
| *Approximate significance of smooth terms* | **edf** | **Ref.df** | **F** | **p-value** |
| s(age) | 1 | 1 | 9.22 | 0.003 |
| ti(age, mass) | 4.11 | 4.11 | 6.02 | <0.0001 |

**Supplementary Figure S11.** Generalized Additive Mixed Model showing duration (minutes) of trips taken by both male and female GPS-tracked Antarctic fur seal pups in relation to pup age and mass (221 trips taken by 13 female pups and 300 trips taken by 16 male pups). Rugs (tick marks inside plot) indicate locations of all data points.

**
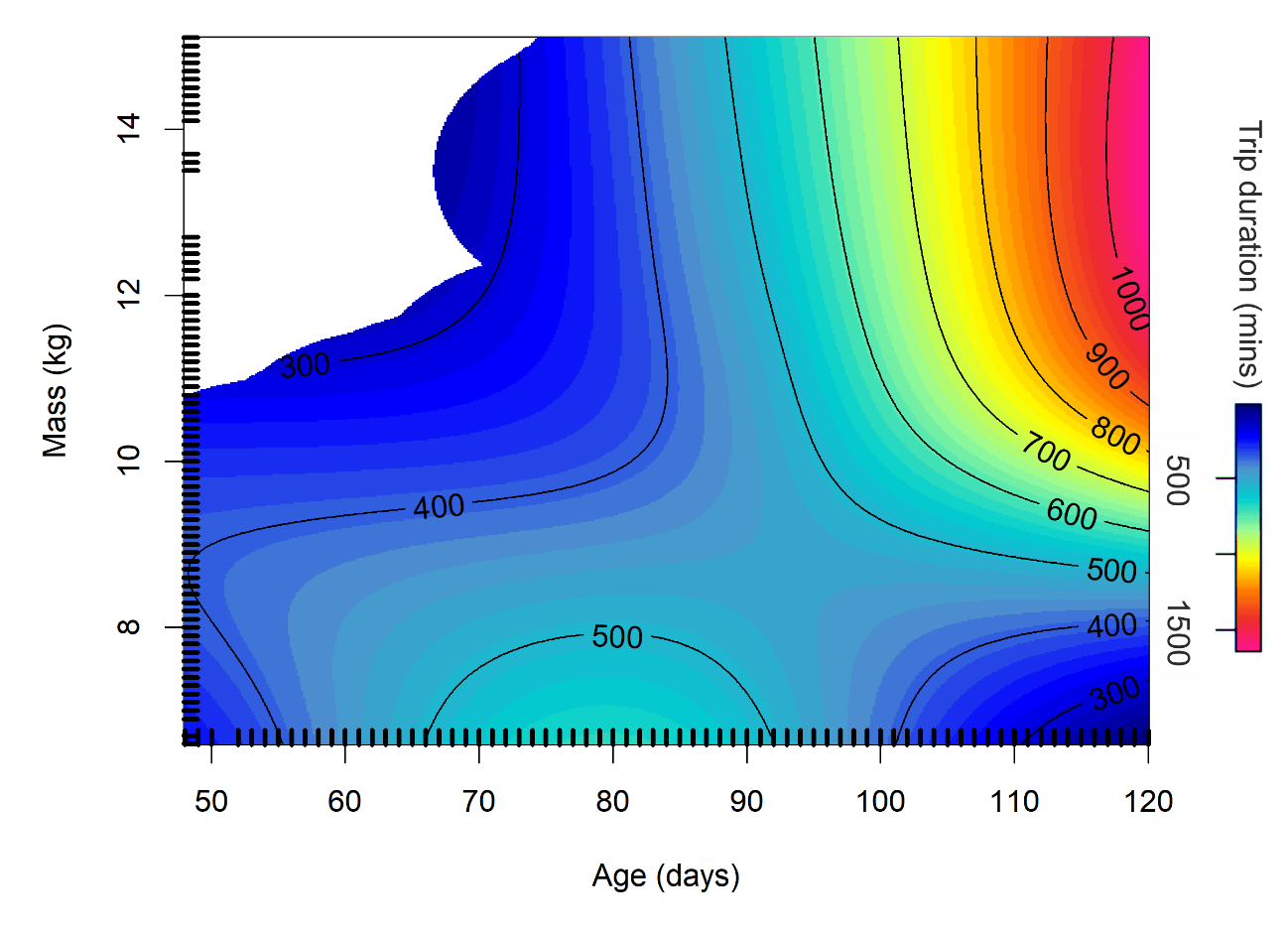
**

**Supplementary Table S12.** Model comparisons to study the effects of sex, smooth function of mass, smooth function of age, and their interactions on the proportion of time that trips of GPS-tracked Antarctic fur seal pups occurred during the night. AIC: Akaike’s information criterion; ΔAIC: difference in AIC between candidate model and best-fit model; R^2^: proportion of variance explained by the predictors; n (N) number of observations of the response variable and number of individuals respectively. Model parameters are shown for the best-fit model.

| **Predictors** | **AIC** | **ΔAIC** | **R^2^** | **n (N)** |
| --- | --- | --- | --- | --- |
| sex + s(age) | 1562.7 | 0 | 0.0279 | 521 (29) |
| sex + s(mass) | 1633.7 | 71.0 | 0.012 | 521 (29) |
| s(age) + s(mass by sex) + ti(age, mass by sex) | 1664.2 | 398.5 | 0.0163 | 521 (29) |
| s(age) | 1949.8 | 387.1 | 0.011 | 521 (29) |
| s(mass) | 1966.2 | 403.5 | 0.000395 | 521 (29) |
| s(mass) + s(mass by sex) | 1970.7 | 408.0 | -0.0049 | 521 (29) |
|  |  |  |  |  |
| *Parametric coefficients* | **Value** | **SE** | **t-value** | **p-value** |
| Intercept | -0.32 | 0.08 | -3.79 | 0.0002 |
| sex (male) | -0.31 | 0.11 | -2.76 | 0.006 |
| *Approximate significance of smooth terms* | **edf** | **Ref.df** | **F** | **p-value** |
| s(age) | 1 | 1 | 8.51 | 0.004 |

**Supplementary Figure S13.** Generalized Additive Mixed Model (with standard error) showing proportion of time that GPS-tracked pup trips occurred during the night in relation to pup age, based on 221 trips taken by 13 female pups (red) and 300 trips taken by 16 male pups (blue). Points indicate proportion of time trips occurred during the night each 24 hrs by individuals, line indicates modeled average and shaded area indicates standard error.

**
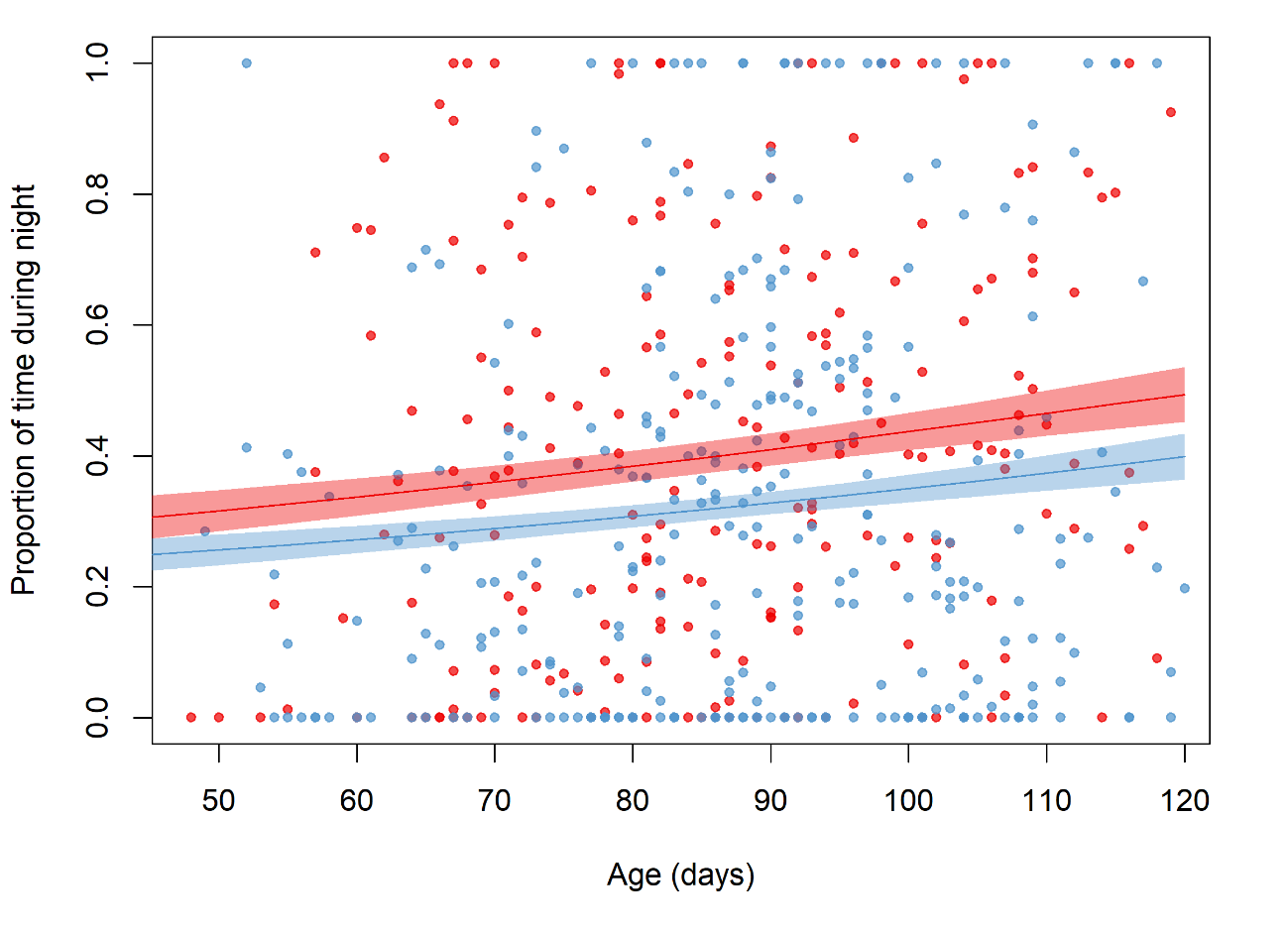
**
